# Can Taichi Reshape the Brain? A Brain Morphometry Study

**DOI:** 10.1371/journal.pone.0061038

**Published:** 2013-04-09

**Authors:** Gao-Xia Wei, Ting Xu, Feng-Mei Fan, Hao-Ming Dong, Li-Li Jiang, Hui-Jie Li, Zhi Yang, Jing Luo, Xi-Nian Zuo

**Affiliations:** 1 Key Laboratory of Behavioral Science, Laboratory for Functional Connectome and Development, Magnetic Resonance Imaging Research Center, Institute of Psychology, Chinese Academy of Sciences, Beijing, China; 2 Key Laboratory of Mental Health, Institute of Psychology, Chinese Academy of Sciences, Beijing, China; 3 Psychiatry Research Center, Beijing Huilongguan Hospital, Beijing, China; 4 Beijing Key Laboratory of Learning and Cognition, Department of Psychology, Capital Normal University, Beijing, China; 5 University of Chinese Academy of Sciences, Beijing, China; Beijing Normal University, China

## Abstract

Although research has provided abundant evidence for Taichi-induced improvements in psychological and physiological well-being, little is known about possible links to brain structure of Taichi practice. Using high-resolution MRI of 22 Tai Chi Chuan (TCC) practitioners and 18 controls matched for age, sex and education, we set out to examine the underlying anatomical correlates of long-term Taichi practice at two different levels of regional specificity. For this purpose, parcel-wise and vertex-wise analyses were employed to quantify the difference between TCC practitioners and the controls based on cortical surface reconstruction. We also adopted the Attention Network Test (ANT) to explore the effect of TCC on executive control. TCC practitioners, compared with controls, showed significantly thicker cortex in precentral gyrus, insula sulcus and middle frontal sulcus in the right hemisphere and superior temporal gyrus and medial occipito-temporal sulcus and lingual sulcus in the left hemisphere. Moreover, we found that thicker cortex in left medial occipito-temporal sulcus and lingual sulcus was associated with greater intensity of TCC practice. These findings indicate that long-term TCC practice could induce regional structural change and also suggest TCC might share similar patterns of neural correlates with meditation and aerobic exercise.

## Introduction

Inactivity is a growing public health concern. About 74% of adults in the United States do not meet the recommended guideline of at least 30 minutes of moderate-intensity physical activity on most days of the week [1]. In developing countries such as China, the rate of sedentary lifestyle seems higher, at 82.1% Chinese adults did not participate in regular physical exercise [2]. This issue is undoubtedly associated with a number of physical (for example, cardiovascular disease, colon and breast cancer, and obesity) and mental (for example, depression and anxiety) disorders [3]. It is also possible that human evolution, which has been shaped by an active lifestyle, will be maladapted by the sedentary behaviors of today [4], [5]. Additionally, economic cost of this sedentary lifestyle brings about enormous social burden in both developed and developing countries. Therefore, highlighting the importance of promoting physical activity across the lifespan could have great implications for improving health and function in individuals, while also reducing the health and economic burden placed on society. However, there are still large numbers of people still keep sedentary during their daily life even when they have full knowledge of the importance of physical activity. It is shown that environmental factors such as lack of fitness equipment, not enough activity space, and subjective factors such as being afraid of injury, contribute to plenty of dropouts during physical exercise [6]. So it is important to select the appropriate exercise type to engage in it when individuals determined to start exercise program.

Tai Chi Chuan (TCC) is a form of mind and body exercise originating from ancient China with a long and rich history based on ancient Chinese Tao philosophy. It can be practiced without special facilities or expensive equipment and can be performed either individually or in groups. Most importantly, because of its low- to- moderate intensity characteristics and slow and relaxed nature, TCC is suited for persons of all ages, including both older adults and individuals with chronic diseases [7]. In view of these merits, it has grown in popularity and spread rapidly over more than 50 countries currently since it was developed into short 24 forms/movements in 1956. As estimated, almost 150 million people practice TCC around the world for its physical health benefits [8], [9] including increasing muscle strength [10], flexibility [11], and balance and motor control [12], reducing risks of falling [7], deceasing factors such as pain [13], blood pressure, Parkinson disease symptoms [14] and cardiovascular diseases (CVDS) symptoms [15]. Additionally, some studies highlight the mental benefits that practicing TCC may achieve when used as an intervention tool, such as its positive effects on mood [16], self-efficacy [15], stress [17], [18] and quality of life [19]. There has been a proliferation of research interest on the effect of TCC on physical, mental and social function over the past decades (see the reviews [7], [11], [18], [20]–[22]. Of note, TCC, as a kind of complex movements, has also been proven to have positive effect on executive function among these benefits in several studies. For instance, Matthews and Williams [23] evaluated the effects of a TCC program on cognitive performance in older adults and found TCC has benefits for the tasks involved in executive function. Similar results have been obtained by Black et al. [24] using an experimental design, which revealed that participants in the TCC program experienced a significant improvement in tasks including components of executive function from baseline to 12 months.

At first glance, this impressive range of topics bears ample testimony to a thriving field. On closer inspection, an important issue arises. This welcome trend is evident in the abundance of recent studies mainly on the effects of TCC on the individual's behavior. However, the potential neural mechanism underlying the behavioral effects induced by practicing TCC has received surprisingly little attention. To date, it is unknown which structural brain changes are associated with TCC practice, not to mention the neural correlates of behavioral change, such as executive function induced by TCC training. Accordingly, we compared groups of highly experienced TCC practitioners and healthy control non TCC practitioners to investigate whether brain structural difference existed between the two groups. We expected brain structural changes to be correlated with experience of practicing TCC and with the performance in executive function.

## Methods

### Ethics statement

Written informed consent from the participants was obtained, and the study “the neural correlates of the effect of Taichi on the mental health” was approved by the Institutional Review Board of the Institute of Psychology, Chinese Academy of Sciences and was performed in accordance with the ethical standards laid down in the 1964 Declaration of Helsinki. The ethics committee specifically approved all of the procedures of this study. Before the scans were taken, all subjects brought the volunteer screening forms to the Institute of Psychology, Chinese Academy of Sciences to exclude any subjects who had a history of hearing or vision problems, physical injury, seizures, metal implants, and head trauma with loss of consciousness, or pregnancy.

### Participants

The sample consisted of two groups. The first group was composed of 22 (7 males) experienced TCC practitioners (age: 52±6 years) recruited from local TCC activity centers in Beijing. On average, participants had 14±8 years of TCC experience, which is an indicator of duration of practice. The practice frequency (practice times per week) and the duration each session were also obtained to account the total hours to practice TCC per week, which is regarded as the intensity of TCC practice. TCC styles mainly included Yang, Wu, Sun and modified Chen. The length of formal TCC practice ranged from 30 to 90 minutes each session, with the majority of TCC practitioners (85%) having daily sessions. Twenty control participants (age: 54±6 years) with no physical exercise, yoga or meditation experience were recruited in this study. Two controls were excluded from the control group who showed macroscopic cerebral abnormalities of cerebral infarction without clinical significance following the consistent diagnosis of reading by two radiologists. The final sample included 22 active TCC practitioners and 18 controls matched for sex, age, race (both groups 100% Han) and years of education (Table 1).

**Table 1:** Participant characteristics.

	Age (years)	Gender	Education (years)	Practice Duration (years)	Practice Intensity (hours/week)	Intracranial Volume (ICV) (mm^3^)
**TCC**	52±6	7 M; 15 F	12±3	14±8	11±3	1165183±221749
**Controls**	54±6	8 M; 10F	12±3	—	—	1114497±170647

### Behaviour tests

Before MRI sessions, the participants completed an Attention Network Test (ANT), which is considered as the flanker test to measure different behavioral aspects of attention including alerting, orienting and executive attention based on the Attention Network theory [25]. Participants were seated in 65 cm front of a computer screen. Stimuli were presented and responses were collected with E-prime Software 2.0. Participants were instructed to respond as fast and accurately as possible to a target stimulus that was presented in the center of a horizontal row with five stimuli. The target stimulus was an arrow pointing either to the left or to the right and was flanked by two flanker stimuli on each side. Secondly, participants were instructed to press the left mouse button with their left thumb or the right mouse button with their right thumb as fast as possible when the target arrow pointed to the left or right, respectively. The four surrounding flanker stimuli were all arrows pointing in the same or the opposite direction of the target stimulus or were just neutral stripes. The condition in which all five arrows pointed in the same direction was called congruent target condition. The condition in which the flanker arrows pointed in the direction opposite to the target arrow was named the incongruent target condition. The condition when the four flanker stimuli were stripes was called the neutral target condition. The target stimulus and the flanker stimuli were presented at a visual angle of 1.1 degree above or below a fixation cross presented in the middle of the screen.

The target stimulus could be cued in four different ways. In the first cueing condition, an asterisk was presented at the location of the fixation cross (center cue condition) and the target configuration was presented above or below the center of the screen, with equal probability. In the second cueing condition, two asterisks were presented (double condition); the two asterisks were presented at the fixed location of 1.1 degree of visual angle above and below the center of the screen. Since the cue appeared 500 ms before target onset, the cue provided information on the timing of the target stimulus. In the third cueing condition, an asterisk was presented at the future location of the target stimulus above or below the center of the screen (spatial cue condition). In this case, participants were informed both on the timing and the location of the target configuration. In the fourth cueing condition, no cue was given and, as a consequence, participants had no information about the timing and the location of the upcoming target symbol.

**Figure 1:**
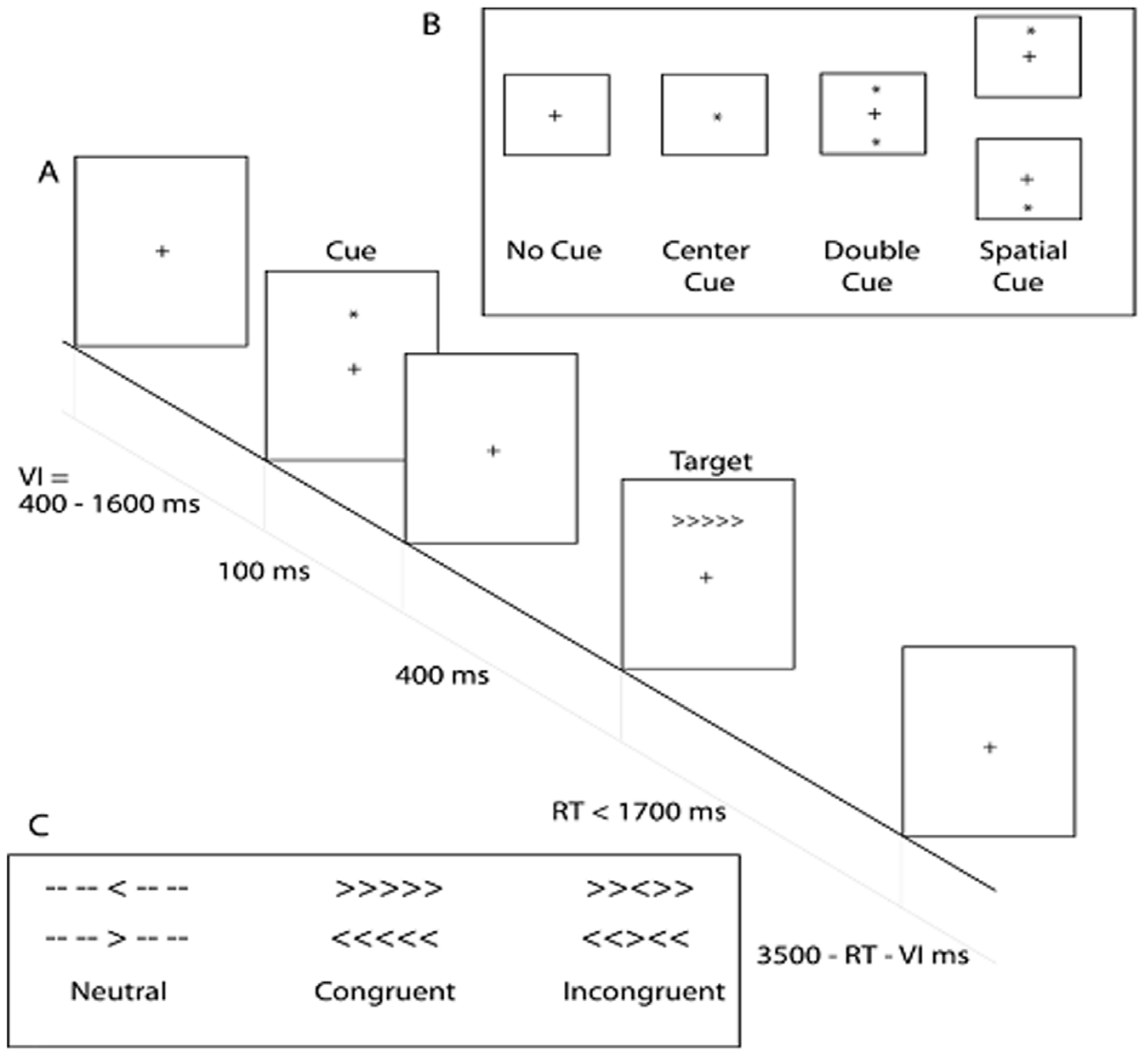
An illustration on the behavioral tests including three stages. During a variable interval (VI = 400–1600 ms), a central fixation cross is presented and the participant is instructed to look at it. A cue then is presented for 100 ms. The four cue conditions are shown: No Cue, Central Cue, Double Cue, and Spatial Cue. Following the cue presentation, a central fixation cross is displayed and is followed by the target stimulus. The three different target configurations are shown: the Neutral, Congruent and Incongruent target configurations. The target is displayed until the participant responds with a maximum of 1700 ms. In case of the reaction time shorter than 1700 ms, the stimulus is replaced by the central fixation cross.

The attention network test consisted of one training block with 24 trials and three test blocks with 96 trials each. After the first two blocks, participants took a short break before starting the next one. A single trial consisted of the following: during a variable interval (VI, see Figure 1), ranging from 400 ms to 1600 ms, a fixation cross was presented in the middle of the screen. Then, depending on the cure condition, a cue could be presented for 100 ms. Thereafter, a central fixation was presented for 400 ms, followed by the target stimulus, which was presented for 1700 ms, or shorter if a response was given within 1700 ms. Finally, a fixation cross was presented during a variable delay. The length of this delay was determined by subtracting the reaction time plus 400 ms from the constant trial duration that was 3500 ms. All 12 combinations of cueing (4) and target (3) conditions were presented in random order within each block. Both reaction time and error scores were measured for each condition. Only half of the control group and TCC group participated in this test because of time limit.

### MRI acquisition

High-resolution anatomical images of the whole brain were acquired on a 3T Trio system (Siemens, Erlangen, Germany) in Beijing Normal University with a 12-channel head matrix coil using a magnetisation-prepared rapid-acquisition gradient echo (MPRAGE) sequence (TR = 2530 ms, TE = 3.39 ms, flip angle = 7 degrees, slice thickness = 1.33 mm, FOV = 256 mm, 256×256 matrix). The scan time for the T1-weighted sequence was 8 minutes and 6 seconds. Brain images were reconstructed and visually checked for major artefacts (e.g. motion, ringing, wrap around and neurological abnormalities) before further image processing.

### MRI preprocessing and surface reconstruction

Structural image processing was conducted with FreeSurfer (version 5.1), which is integrated as part of the pipeline of Connectome Computation System (CCS: http://lfcd.psych.ac.cn/ccs.html) [26]. Specifically, individual MR images were first denoised by using a spatially adaptive non-local means filter [27], [28] and corrected for intensity variations due to MR inhomogeneities [29]. Brain tissues were then extracted using a hybrid watershed/surface deformation procedure [30] and automatically segmented into the cerebrospinal fluid (CSF), white matter (WM) and deep gray matter (GM) volumetric structures [29]. Cutting planes were then computed to disconnect the two hemispheres and subcortical structures [29], and the interior holes of the segmentation were filled by a connected component analysis [29]. A smooth representation of the GM-WM interface (i.e., white surface) and GM-CSF interface (i.e., pial surface) was produced by transforming a tessellation of a triangular mesh over the GM-WM boundary of each hemispheric volume [29], which was further corrected for topological defects in the surface to achieve a spherical topology [31], [32]. Finally, each subject's surface mesh was inflated into a sphere to compute a smooth, invertible deformation of the resulting spherical mesh to a common spherical coordinate system that aligned the cortical folding patterns across subjects [29], [33].

### Quality assurance

During the preprocessing, CCS generated various figures to guide three quality assurances (QAs) on brain extraction, surface reconstruction and anatomical images registration (http://lfcd.psych.ac.cn/ccs/QC.html). Specifically, the quality of the brain extraction and intensity bias correction must be visually assessed and manually corrected if the procedure failed. The brain tissue segmentation and brain surface reconstruction were also visually checked to ensure a good quality. Two researchers (F.M.F and H.M.D) carried out the QAs to double check the quality matching the criteria. No subjects failed to pass the quality check.

### Brain morphometry computation

Computation of cortical thickness, surface area and volume are completed by the *recon-all* command. Specifically, for each vertex on the white matter surface, the shortest distance to the pial surface is first computed. For the point on the pial surface, the shortest distance to the white matter surface is calculated. Finally, the cortical thickness at that location of the vertex is the mean of these two values [34]. This measure of cortical thickness has been demonstrated to show good test–retest reliability across time, scanner manufacturers and across field strengths [35]. Cortical surface area was computed as the total area of the triangles connected to a vertex [34], [36]. This measure is in agreement with surface area derived from post-mortem studies and has been validated on several brain phantoms and compared with other surface-based analysis packages [37]–[39]. Cortical volume is calculated as the product of the cortical thickness and surface area for each vertex. All individual maps of cortical thickness and surface area were generated in the native space and then transferred into the *fsaverage* standard spherical surface.

### Parcel-wise analysis

The cortical surface was parcellated into 74 and 34 parcellation elements (parcels) for each hemisphere defined by the Desikan-Killiany atlas [40], [41] and Destrieux atlas [42] to explore the large-scale structural changes, respectively. The mean of vertex-wise cortical thickness, volume and surface area were then estimated for each parcel of all participants. In addition, the subcortical structure was segmented into 17 regions providing 8 regions (Amygdala, Caudate, Hippocampus, Accumbens-area, Pallidum, Putamen, Thalamus-Proper, Cerebellum-Cortex) in each hemisphere and Brainstem. The volume of each subcortical region was calculated.

For each parcel, group differences in cortical morphological indices between TCC group and control group were assessed in SPSS 19.0 using analysis of covariance (ANCOVA). The age, sex, education and intracranial volume (ICV) were modelled as covariates. The statistical significance level for group differences in cortical thickness and surface area was corrected to account for multiple comparisons with Bonferroni approach (i.e., *p*<0.05/N) where N is the number of parcels in one hemisphere (34 for Desikan-Killiany atlas and 74 for Destrieux atlas). Of note, the number of parcels for volume-based analysis is 43 and 83 for these two atlases including the 9 additional subcortical parcels, respectively. In order to further examine whether the difference in regional cortical morphology was associated with TCC practice, we conducted partial correlation analyses between these two variables by modeling sex, age, education and ICV as covariates. Finally, correlation analysis was conducted between the ANT performance and structural measures in both groups for these brain regions showing significant morphometrical changes.

### Vertex-wise analysis

Individual morphometry surface (registered to *fsaverage*) maps (thickness, area and volume) were first spatially smoothed using a Gaussian filter of 10 mm FWHM to improve the inter-individual anatomical correspondences. For each vertex, a general linear model (GLM) was carried out to examine the cortical morphometry differences between TCC and controls. All these GLMs include two discrete factors (sex and group) and three continuous factors (age, education and ICV) where the group is the variable of interest. We then tested the effect of Group on the three morphometric measures. To account for multiple comparisons, these surface GLMs were corrected at cluster-level (p<0.05) by using random field theory as implemented in Matlab-based functions in FreeSurfer [43].

## Results

### Demographic data and behaviour performance

**Figure 2:**
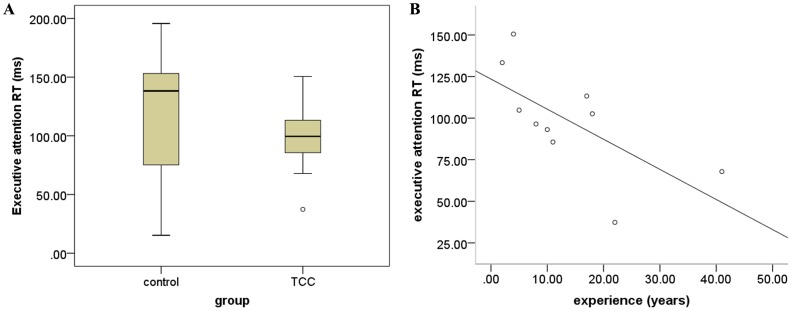
Group differences in behavioral task performance. A) group differnces in performance of executive attention between TCC group and control group; B) scatter plot of the association between performance of executive attention and TCC experience (years of practice).

The two-sample T-tests revealed no significant difference in age (*t* (38) = 1.149; *p* = 0.258) and education (*t* (38) = −0.435; *p* = 0.666). The two groups also did not differ in ICV observed (*t* (38) = 0.817; *p* = 0.419). For the reaction time of ANT, TCC group exhibited shorter mean reaction time relative to the control group in terms of executive function performance (Figure 2A), although this difference was not significant (*t* (18) = 1.227; *p* = 0.236). In addition, no significant group difference in accuracy of ANT performance was detected (control group: 99.0%±0.008; TCC group: 99.5%±0.006; *t* (18) = −1.421, *p* = 0.173). Correlation analysis for TCC group showed that the performance of executive attention was negatively correlated with TCC experience (*r* = −0.659; *p* = 0.038) (Figure 2B).

### Group differences in cortical morphology and subcortical morphology

**Figure 3:**
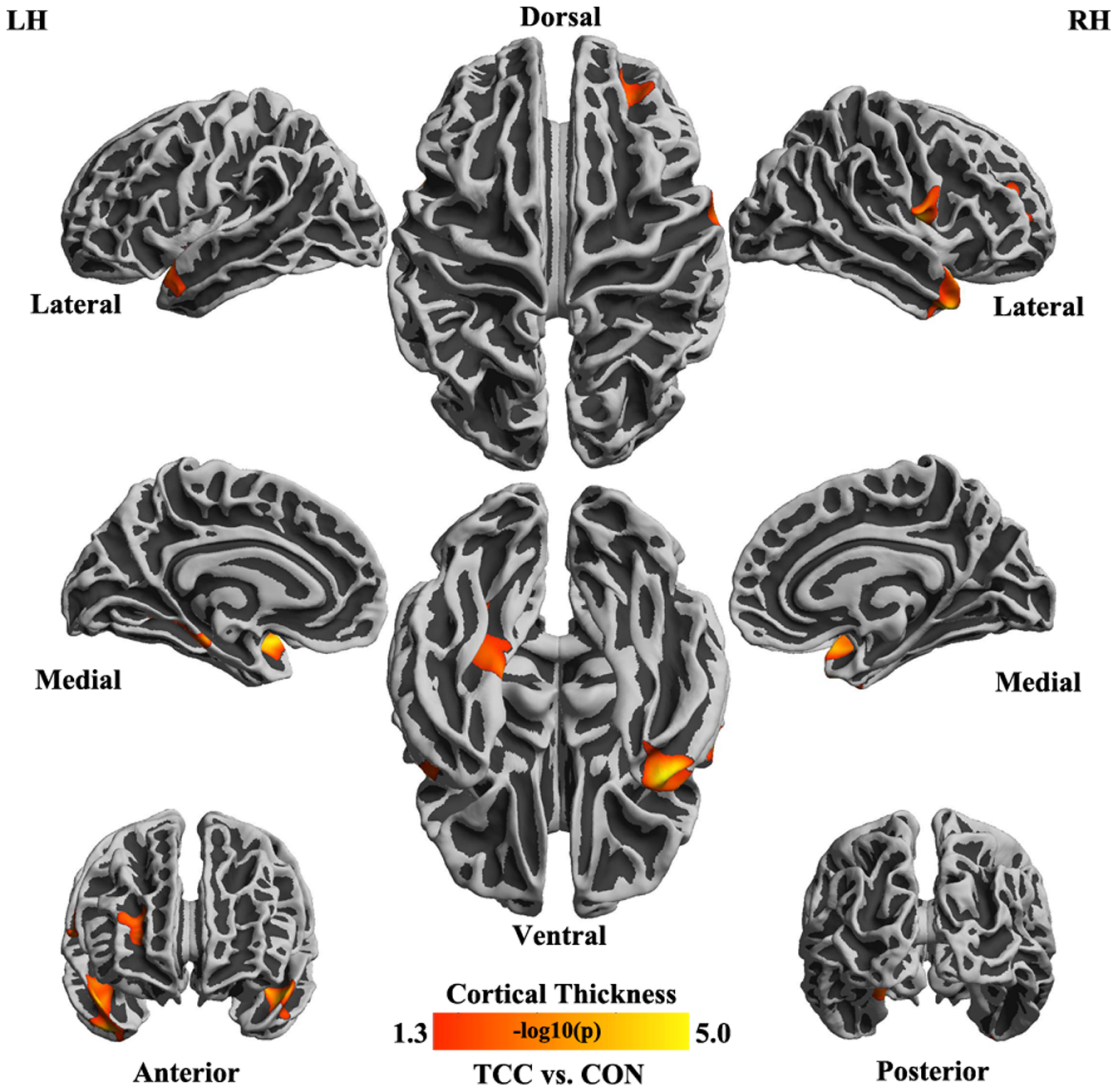
Cortical regions thicker in TCC practitioners than in controls. Statistical map depicting between-group differences in thickness at each point on the cortical surface overlaid on the inflated average brain. All points meeting a *p*<0.05 (corrected) threshold are displayed to better illustrate the anatomic extent of the areas and relative specificity of the findings.

For all parcels derived from both Desikan-Killiany atlas and Destrieux atlas, we did not detect any differences in thickness, area and volume between the two groups. As for subcortical parcels, no significant difference in their volumes was observed between two groups. In vertex-wise analyses, we observed significantly thicker cortex in TCC group compared to control group: superior temporal gyrus (*t* (38) = −3.699, *p* = 0.001), medial occipito-temporal sulcus and lingual sulcus (*t* (38) = −3.821, *p* = 0.001) in left hemisphere and the inferior segment of the circular sulcus of the insula (*t* (38) = −4.202, *p* = 0.0002), precentral gyrus (*t* (38) = −4.280, *p* = 0.0001) and middle frontal sulcus (part of dorsal lateral prefrontal cortex-DLPFC) (*t* (38) = −2.512, *p* = 0.017) in right hemisphere (Table 2). Beyond the cortical thickness, there was no any significant change of cortical volume and surface area detectable in TCC practitioners relative to the controls (Figure 3).

**Table 2:** Thicker cortex in TCC group compared with control group.

Brain region	Brodmann Area	Area Size (mm^2^)	Number of Vertex	Talairach Coordinates (Peak Vertex)			*P-value*
**Left hemisphere**							
**G_temp_sup-Plan_polar**	38	591.77	1197	44.1	7.4	−20.6	0.001
**S_oc-temp_med_and_Lingual**	36	402.45	909	20.7	−40.0	−6.5	0.001
**Right hemisphere**							
**S_circular_insula_inf**	20	1085.63	2054	42.2	−2.2	−17.8	0.0002
**G_precentral**	44	447.49	1036	59.2	5.0	21.2	0.0001
**S_front_middle**	9	420.15	712	23.2	42.4	19.4	0.017

G_temp_sup-Plan_polar  =  planum polare of the superior temporal gyrus; S_oc-temp_med_and_Lingual  =  medial occipito-temporal sulcus (collateral sulcus) and lingual sulcus; S_circular_insula_inf  =  inferior segment of the circular sulcus of the insula; G_precentral  =  precentral gyrus; S_front_middle  =  middle frontal sulcus.

### Correlations between TCC experience and cortical morphology

**Figure 4:**
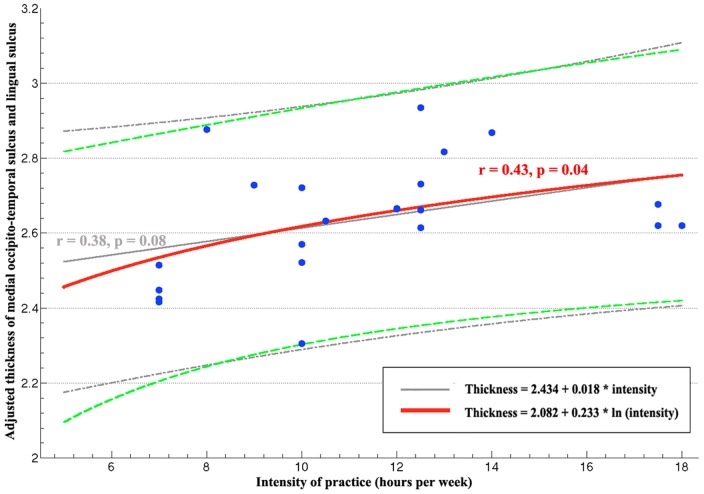
Scatter plot of cortical thickness in TCC group. The red line represented the correlation between logarithm transformed intensity and cortical thickness (adjusted by sex, age, education and ICV) in left medial occipito-temporal sulcus and lingual sulcus. The green dotted lines indicated its 95% confidence intervals. The linear correlation between intensity and the cortical thickness in this region are displayed in grey line with its confidence intervals as dotted.

To examine whether the cortical thickness of each of these five regions showing group differences correlated with intensity of TCC practice, we calculated partial correlations controlling for the sex, age, education and ICV. We detected a trend of a positive correlation between the thickness of left medial occipito-temporal sulcus and lingual sulcus and the intensity of TCC practice (*r* = 0.38, *p* = 0.084). We further noticed that the values of intensity of TCC practice here were marginally nonnormally distributed (Shapiro-Wilk Test, *p* = 0.056). The effect of TCC practice on brain morphology may follow a non-linear relationship as indicated in previous studies of TCC on other physical properties [44], [45]. Specifically, the change of relevant cortical thickness can be slower than increasing speed of the intensity of TCC practice. Accordingly, we performed a natural logarithm transform on the original score of intensity of TCC practice. The normality of intensity scores of TCC practice was improved with this transformation (Shapiro-Wilk test, *p* = 0.119). Thus, the thickness of left medial occipito-temporal sulcus and lingual sulcus significantly correlated with log-transformed intensity of practice (*r* = 0.43, *p* = 0.045) (Figure 4), while no other significant correlation were detected based upon the transformed intensity for other four clusters.

### Correlations between ANT and cortical morphology

For each of the five clusters showing differences in cortical thickness between TCC experts and controls, we performed partial correlation analysis between ANT and its cortical thickness in both TCC group and control group. The results indicated that the reaction time of ANT was positively correlated with cortical thickness of left superior temporal gyrus (*r* = 0.929, *p* = 0.007) in TCC group, whereas this relationship was not detectable in control group.

## Discussion

To the best of our knowledge, the current study is the first to systematically explore the brain morphometry of TCC practitioners. We observed TCC practitioners had thicker cortical thickness in left superior temporal gyrus, left medial occipito-temporal sulcus and lingual sulcus, right inferior segment of the circular sulcus of the insula, right precentral gyrus and right middle frontal sulcus (part of dorsolateral prefrontal cortex-DLPFC) compared to the controls. The thickness of medial occipito-temporal sulcus and lingual sulcus was detected to have a trend toward positive correlation with the intensity of TCC practice, providing evidence that long-term TCC practitioners have structural alterations in grey matter, which is possibly related to regular exercise. The consistency of our results with brain studies of meditation and aerobic exercise, leads us to speculate that the underlying mechanism of TCC training effects on brain structure might overlap with those other activities.

The most interesting findings of the present study were that the differences in these brain regions were remarkably consistent with previous studies of brain structural measures in meditation practice [46]–[48]. The first study in morphological differences in meditators by Lazar et al. [46] compared cortical thickness of 20 insight meditation practitioners with 9 years of average meditation experience and 15 matched controls. Greater thickness was found in the left superior temporal gyrus, the right anterior insula, right prefrontal cortex, right middle and superior frontal sulci (BA 9) in meditation practitioners. Holzel et al. [49] found that meditation practitioners had a significantly higher concentration of grey matter in inferior temporal gyrus and right anterior insula in a sample of 20 meditation practitioners relative to 20 controls, which also overlapped with the main results of current study. Moreover, Kang and his colleagues observed that superior frontal cortex, temporal pole and interior temporal cortex were thicker in meditators compared to the controls [50]. Coincidently, these brain regions are very similar with our findings in current study. [1], [51], [52] suggested that other forms of yoga and meditation would likely have a similar impact on cortical structure, although each tradition would be expected to have a slightly different pattern of cortical thickening based on the specific mental exercise involved. Our results showed that TCC group showed greater cortical thickness in some specific regions such as prefrontal cortex and temporal cortex relative to the control group. These brain regions are also reported to be greater in grey matter volume or concentration in neuroimaging studies of aerobic exercise. In fact, a growing body of literature has linked aerobic exercise with these structural changes. Human neuroimaging studies have similar findings which have consistently shown that chronic aerobic exercise could lead to an increasing gray and white volume in the prefrontal cortex of older adults [51]. In addition, greater amounts of physical activity are associated with sparing of prefrontal and temporal brain regions of late adulthood over a nine-year period [53]. Further, medial temporal lobe volumes are larger in higher-fit older adults [53]. Therefore, it possibly provides neuroimaging evidence for a long-standing view that TCC is a form of meditative movement greatly integrating characteristics of meditative practice and aerobic exercise.

Of note, the largest group difference was in the thickness of precentral gyrus. The precentral sulcus, located approximately in primary motor cortex, is responsible for observation and execution of motor tasks and important for highly automatic circuitry because it mediates visuomotor actions [54]. In addition, the final cortical output for already processed movement commands relays signals from premotor cerebral cortical sites to the spinal cord. The significant correlation between the surface area of precentral gyrus and TCC experience indicates that TCC practice might enhance corresponding capability to process motor-related information. According to an arrangement of motor homunculus, the superior part of precentral sulcus is responsible for the somatotopic representation of body parts of hand, arm, trunk, hip and foot. Such bodily regions are often used in practicing TCC movements such as weight-shifting between right and left legs, knee flexion, straight and extended trunk, rotation, and asymmetrical diagonal arm and leg movements with bent knees. Currently, although TCC practice is reported to decrease falling and increase balancing in the elderly in clinical intervention trials, no related brain research has disclosed the underlying neural correlates [55]. We suppose this findings is helpful to explain why practicing TCC can improve balance and prevent falls [56]. However, this inference needs to be tested directly between the falls and anatomical change in the future.

The middle frontal sulcus corresponding approximately to BA9 was also found to have greater cortical thickness in TCC practitioners. This region serves as the highest cortical area responsible for motor planning, organization, and regulation and also plays an important role in the integration of emotion and cognition, and executive function [57], [58]. Recent behavioral studies on TCC indicate that the significant effect of TCC on cognition mainly focuses on executive control (see reviews,[20]). Meanwhile, most studies on meditation reported that DLPFC might be the key region during meditation in structure and function. [46], [59], [60]. Moreover, a large number of studies demonstrate an important role of BA9 in for exercise-related improvement of executive function in adolescents and the elderly [1]. It has been hypothesized that TCC practitioners improved their cognitive processing ability, especially executive function, by controlling breath, focusing on concentration, mindfulness, and inhibiting distractions from surroundings regularly[20].

Right insula is a brain region showing a great between-group difference. Greater right insula grey matter correlates with increased accuracy in the subjective sense of the inner body, and with negative emotional experience [61]. Insula is also believed to process convergent information to produce an emotionally relevant context for sensory experience such as pain, happiness and sadness and integrate information relating to bodily states into higher-order cognitive and emotional processes in functional imaging studies [62]. Our finding further supports the crucial role of the insula for the experience of peacefulness and relaxation during TCC practice.

In the medial occipito-temporal sulcus and lingual sulcus, we not only found the thicker cortex but also the positive correlation with the intensity of practice, indicating potential influences of TCC practice on these regions. The medial occipito-temporal sulcus and lingual sulcus corresponds approximately to BA 36, which is reported to be activated in retrieving spatial information [63], [64]. Neuropsychological and neuroimaging studies suggest BA36 is involved in acquiring spatial information [65]. According to the involvement of muscles in the movement, TCC is classified to be a kind of gross motor skills requiring moving the trunk and limbs by spatial navigation towards oneself, during which practitioners could simultaneously integrate various kinds of spatial information, mainly from proprioception. Therefore, we speculate that the brain region involved in spatial navigation and spatial information acquisition seems to play an important role in performing TCC movements, and may grows thicker after long-term practice.

Although MRI findings clearly suggest that some structural changes are occurring, they are unspecific regarding the underlying cellular events for these structures. Based on some studies ranging from cell level to behaving animals, it is reasonable to assume that some candidate mechanisms such as synaptogenesis [66], [67], changes in neuronal morphology [67] and proliferation of glial cells [23] might contribute to these structural changes during TCC practice. One explanation for these various morphological alternations might relate to the formation of new connections by dendritic spine growth (synaptogenesis) and proliferation of glial cells (gliogenesis), which reflected remodeling of neuronal processes, rather than neurogenesis [68]. This view is also supported by plenty of relevant animal studies. For instance, motor skill learning is found to be associated with synaptogenesis and changes in dendritic spine morphology [66], [67]. In view of our findings in precentral gyrus, an alternative explanation is that TCC experience as a typical form of physical exercise may alter vasculature [69]. It is well documented in animals involved in chronic exercise that exercise increases the vascular volume fraction in rat cortex, especially in motor cortex [70], [71]. As for the neurogenesis, some studies have demonstrated that the changes in the size and number of neurons are a minor factor in MRI changes, particularly those found outside the hippocampus in association with learning [69]. Regarding the underlying cellular mechanism, it still remains unclear to what extent TCC exercise influence cortical thickness. The radial unit hypothesis identifies the cortical column as a fundamental unit of cortical organization. It is suggested that cortical thickness is related to the number or density of cells in a column [72]–[74], which might help to explain the detailed change of thickness in cellular level.

In behavioral testing, we observed significant correlation between practice duration and ANT test, which indicated that executive function of TCC practitioners is associated with TCC experience. Superior temporal gyrus thickness showed a positive correlation with ANT reaction time in our study, which means that the relationship between better performance in ANT test and thinner cortex in these regions might be a sample artifact. In correlation analysis, we observed that the cortical thickness was positively correlated with the intensity of practice instead of practice duration. As a matter of fact, quite a few studies have failed to detect a significant correlation between practice duration (months or years) and brain structures [59], [75]. One reason might result from the inaccuracy of the indicators to reflect experience. Luders suggests that the extrapolations over lengthy periods are subjective rather than precise, although researchers have subject-specific estimates with respect to frequency and length of their practice sessions[76]. Undoubtedly, our findings lend support to his explanation for the inconsistent results [76]. Meanwhile, it implies that it is of great importance to choose an appropriate indicator for determining the actual extent of the individual training.

## Conclusion

Although cross-sectional study cannot rule out the pre-existing difference in brain structures, our findings may suggest the difference in cortical thickness for TCC practitioners might be associated with TCC practice. The underlying neurological mechanism for long-term TCC practice might have similar pattern to cortical morphology associated with meditation and aerobic exercise. Exploration on uses of TCC as one modality of behavioral intervention to maintain and enhance the human brain structure and function is an exciting avenue of research with the potential for a considerable public health yield. At the same time, this result indicates that it is imperative to conduct longitudinal studies aiming to disclose the real causal relationship between the change of brain structures and TCC practice.
